# A Calcifying Odontogenic Cyst With Compound Odontoma in the Maxillary Sinus: A Case Report in a Pediatric Patient

**DOI:** 10.7759/cureus.54679

**Published:** 2024-02-22

**Authors:** Rodrigo A Oliveira, Ana C Maurício, Maria L Sacramento, Jorge Pinheiro, Mariana Moreira

**Affiliations:** 1 Stomatology, Centro Hospitalar Universitário de São João, Porto, PRT; 2 Anatomic Pathology, Centro Hospitalar Universitário de São João, Porto, PRT

**Keywords:** impacted teeth, oral and maxillary pathology, gorlin's cyst, oral histopathology, hybrid odontogenic tumors, calcifying odontogenic cyst, odontoma, odontogenic cyst

## Abstract

Calcifying odontogenic cysts (COCs) exhibit a diverse clinical course, commonly developing between the second and third decades of life, displaying no gender predilection. A 15-year-old female without medical history was under observation for a mixed lesion in the maxilla associated with an impacted tooth. She presented to the emergency department with sudden onset and worsening swelling of the left midface. Radiographic findings in the panoramic radiograph and a CT scan revealed a well-circumscribed mixed lesion localized in the left maxilla, extending into the left maxillary sinus and reaching the orbital floor. After an intercurrent infection of the cyst, the patient was hospitalized, received intravenous antibiotics, and went for surgical intervention under general anesthesia. Lesions that combine histological characteristics of two or more odontogenic tumors or individual cysts in the same location are called hybrid odontogenic lesions. This type of lesion poses a challenge for both pathologists and surgeons, because of its controversial histogenesis and poorly understood clinical evolution. The most common of these lesions are COCs associated with odontoma. Our case represents an exceptionally rare entity among odontogenic cysts.

## Introduction

Odontogenic lesions encompass a diverse group of pathologies classified into odontogenic tumors and cysts of the jaws, according to the latest (fifth edition) of the World Health Organization (WHO) Classification of Head and Neck Tumors 2022 [1]. The calcifying odontogenic cyst (COC) typically emerges between the second and third decades of life, displaying no gender predilection. While its occurrence is nearly equal in the maxilla and mandible, the maxilla exhibits a stronger predilection for the anterior region [1].

First described by Gorlin in 1962, the nomenclature of this lesion has undergone variations, occasionally being referred as the “Gorlin cyst” [2]. Despite its historical classification as a tumor in the 2005 WHO edition, the current classification places it under the cyst category.

The COC is a rare entity, representing less than 1% of all odontogenic cysts [3]. In most cases, it is an intraosseous lesion; however, up to 30% of the cases reported in the literature may manifest as a peripheric lesion, in the gingiva, for example [4].

Radiologically, the COC is characterized by a well-defined unilocular lesion with evident calcifications within the cyst's lumen. Additionally, impacted teeth, root resorption, or tooth displacement may be observed, with up to 24% of cases showing an association with odontomas, more prevalent in younger individuals [3,4].

Histopathologically, the COC features a unilocular cyst with a stratified epithelial lining of varying thickness with palisaded hyperchromatic basal cells and loosely arranged suprabasal cells, similar to ameloblastoma. However, COC features groups of ghost cells in the suprabasal epithelium, which may undergo calcification. These cells have polyhedral morphology and prominent eosinophilic cytoplasm and undergo karyolysis, resulting in a “ghost-like” appearance. Even though these types of cells are characteristic, they can appear in other odontogenic tumors, such as the dentinogenic ghost cell tumor and ghost cell odontogenic carcinoma. To establish this diagnosis, it is essential to have cystic architecture, numerous ghost cells, and, ideally, palisaded hyperchromatic basal cells and dentinoids [1]. Other findings that can be observed in COC are paucicellular eosinophilic dentinoid matrix laid down in the stroma under basal cells, dystrophic calcification, and foci of foreign body giant cells. These cysts may be associated with an odontoma.

## Case presentation

**Figure 1:**
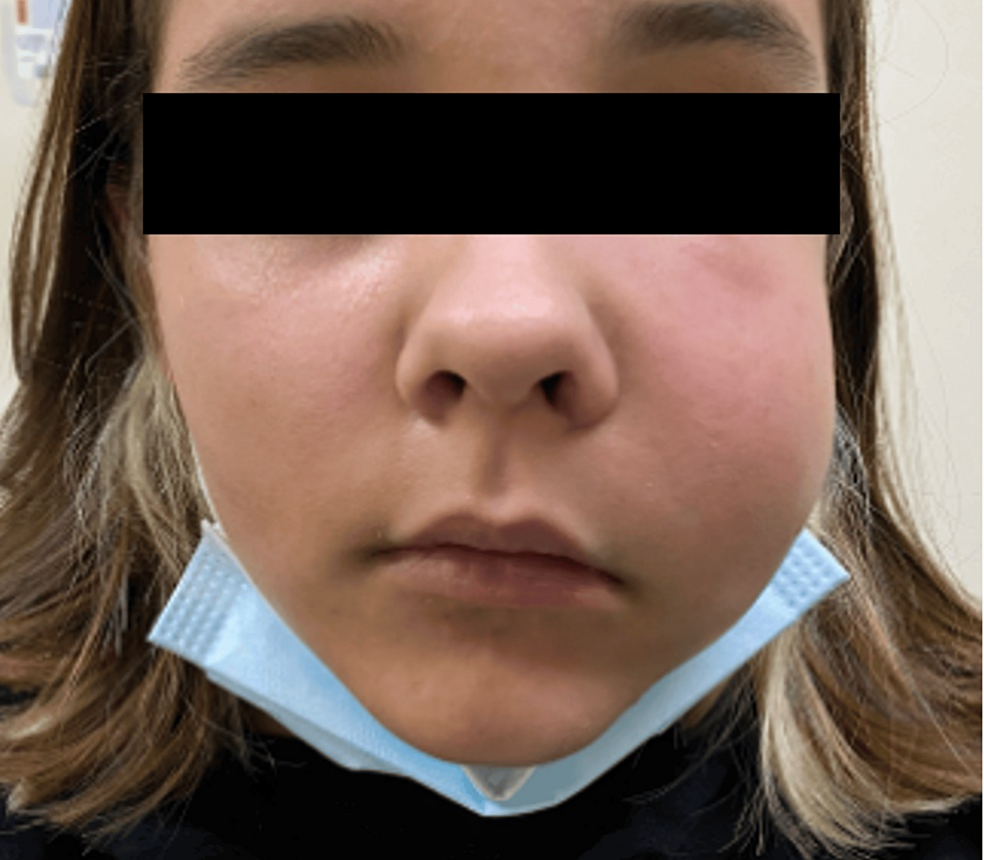
Facial asymmetry at presentation Preseptal cellulitis and swelling of the left midface with skin redness.

A 15-year-old female without significant medical history was under observation for a mixed (radiolucent and radiodense) lesion in the maxilla associated with an impacted tooth. Incisional biopsy confirmed an odontogenic cyst. Three months later, she presented to the emergency department with sudden onset and worsening swelling of the left midface (Figure 1). Initial complaints were toothache, trismus, and intraoral purulent discharge. At the clinical evaluation, the patient presented exuberant edema and redness of the left hemiface, reaching the infraorbital area, intraoral examination revealed edema in the left upper vestibular mucosa with purulent drainage and limited mouth opening. No limitation on eye movements. Blood analyses showed leukocytosis and positive inflammatory markers.

**Figure 2:**
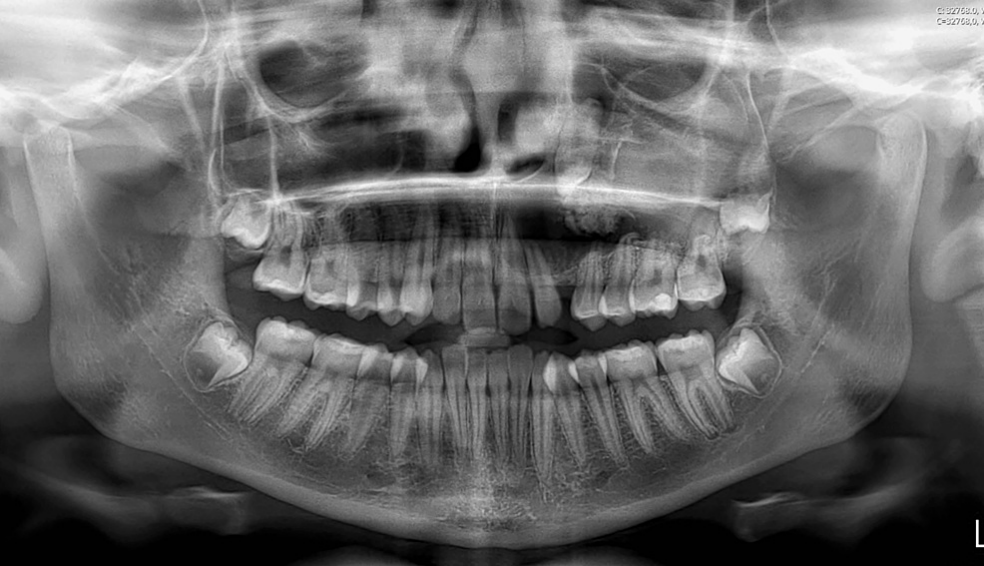
Panoramic radiograph Unilocular radiolucent and radiopaque lesion occupying the entire left maxilla, reaching the left orbit associated with an impacted canine.

**Figure 3:**
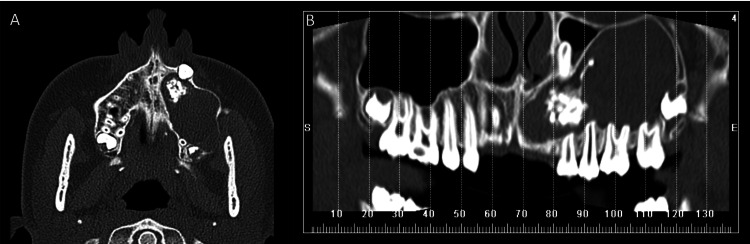
CT scan A: axial view; B: panoramic reconstruction; intraosseous well-defined radiolucent lesion, 6 cm in diameter, with an irregular calcification inside and an impacted tooth next to it. The lesion reaches the left orbital floor and causes root dilaceration and reabsorption of all maxillary teeth.

Radiographic findings in the panoramic radiograph (Figure 2) and CT scan (Figure 3) revealed a well-circumscribed mixed lesion localized in the left maxilla, extending into the maxillary sinus and reaching the orbital floor. Root reabsorption and dilaceration and displacement of adjacent maxillary teeth were observed. Inside the lesion, a permanent canine and a radiopaque irregular lesion were identified.

**Figure 4:**
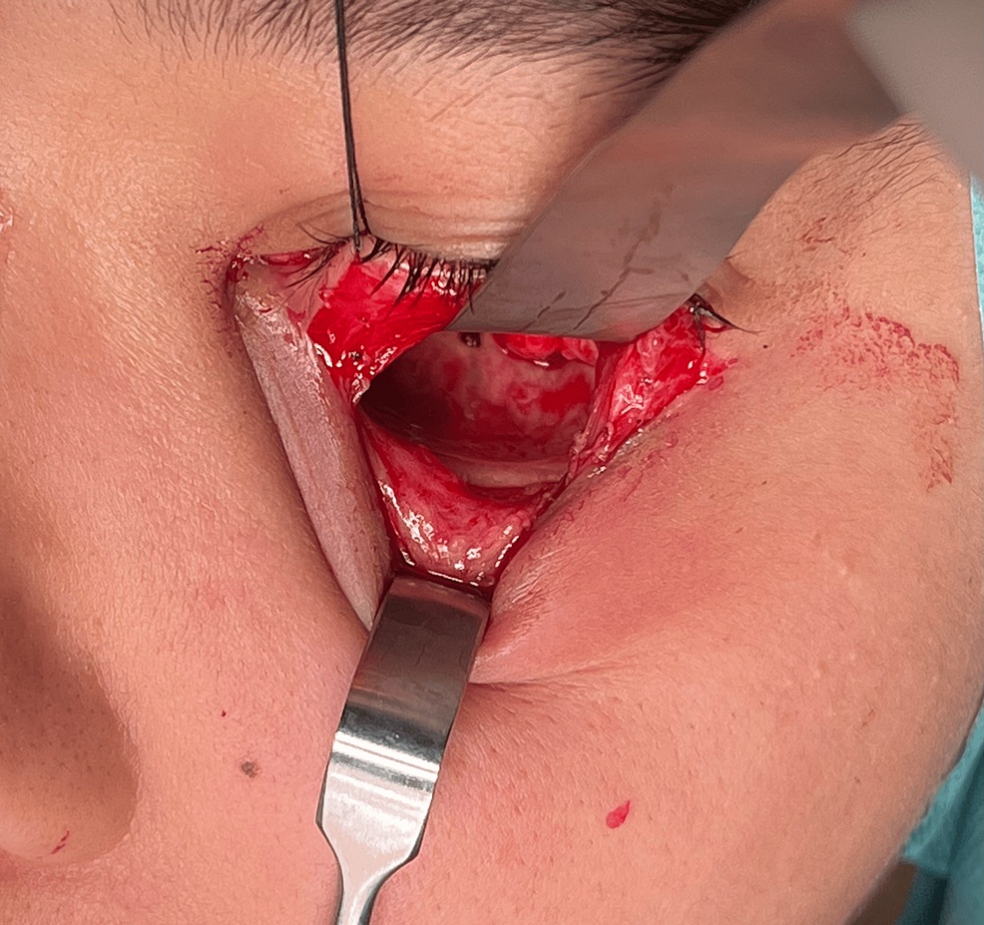
Surgery: orbital floor approach The orbital floor shows neither protuberance nor signs of the lesion.

**Figure 5:**
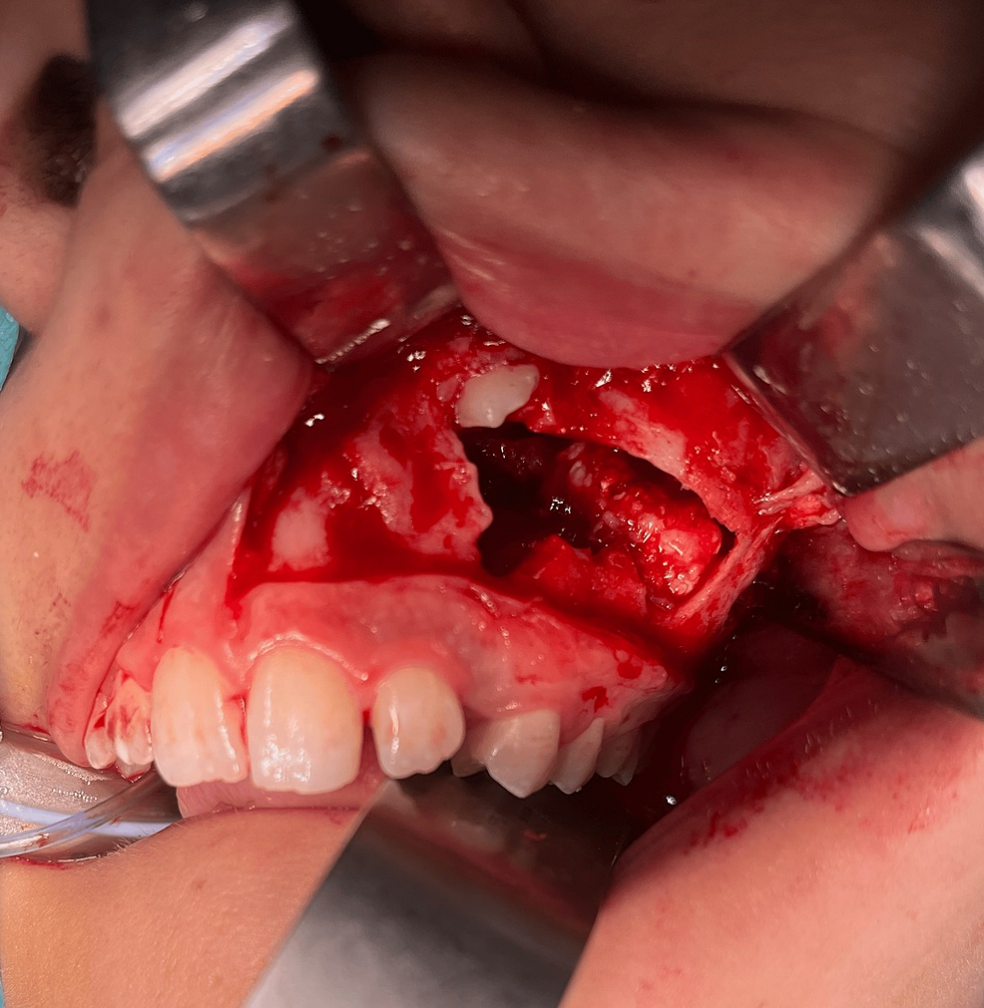
Surgery: Caldwell-Luc approach Maxillary sinus with the lesion inside. It is possible to see the calcifications of the lesion and the canine crown on top.

**Figure 6:**
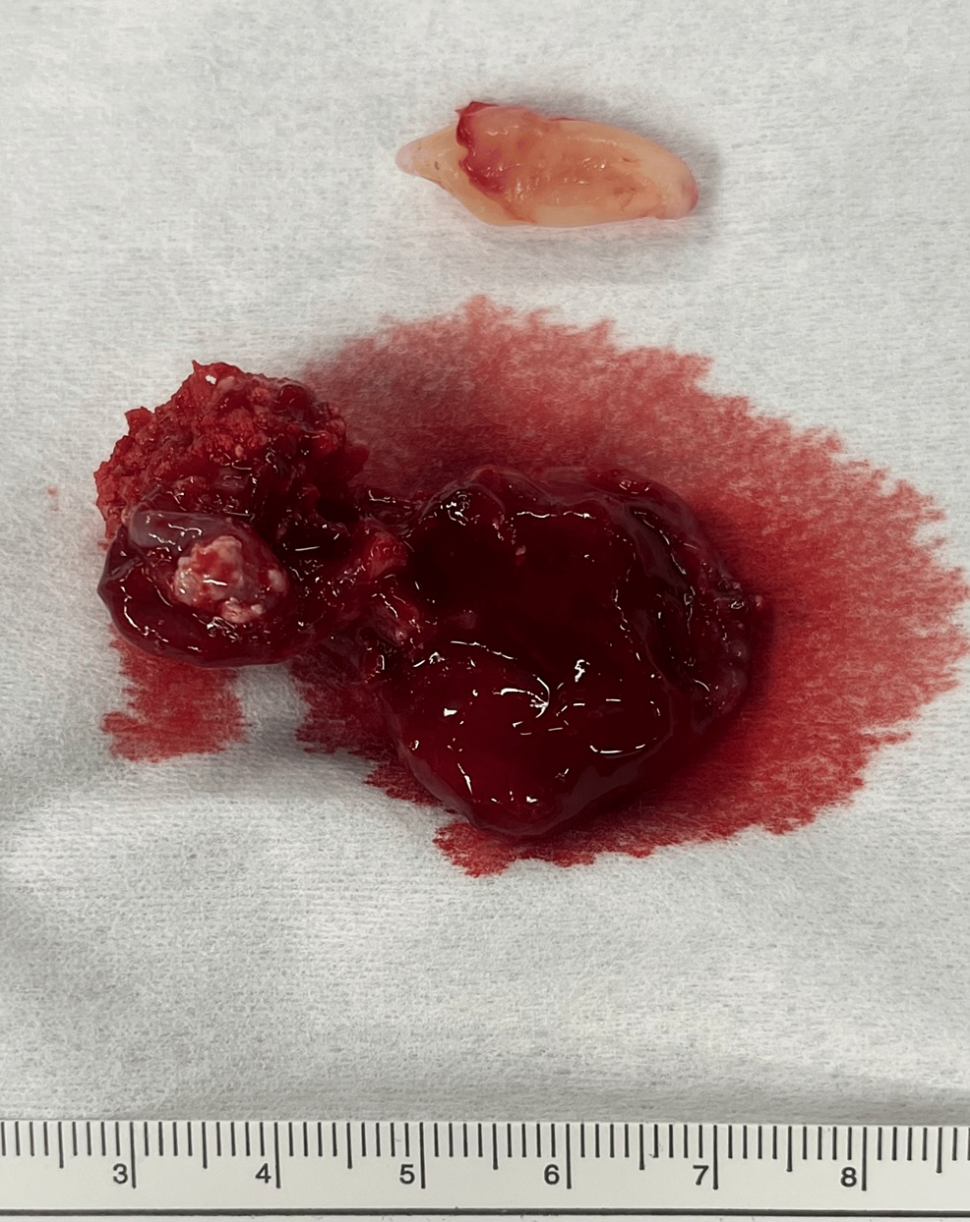
Surgical specimen In the upper part of the image, we can see the impacted canine. At the bottom, there is a lesion of approximately 4.5 cm (showing the hard tissue on the left).

Treatment: After an intercurrent infection of the cyst, the patient was hospitalized. She received intravenous antibiotics (amoxicillin/clavulanic acid) and went for surgical intervention under general anesthesia. A combined approach was done through the orbital floor and intraorally (Caldwell-Luc approach), and a silicon mattress was placed to protect the orbit content during the excision of the lesion (Figure 4). It was completely excised, and the circumferential bone went through curettage (Figure 5). A collagen membrane was fixed to the maxilla with micro screws to close the incisional site, promote bone regeneration, and prevent soft tissue growth into the maxillary sinus. The specimen (Figure 6) was sent for pathological examination.

**Figure 7:**
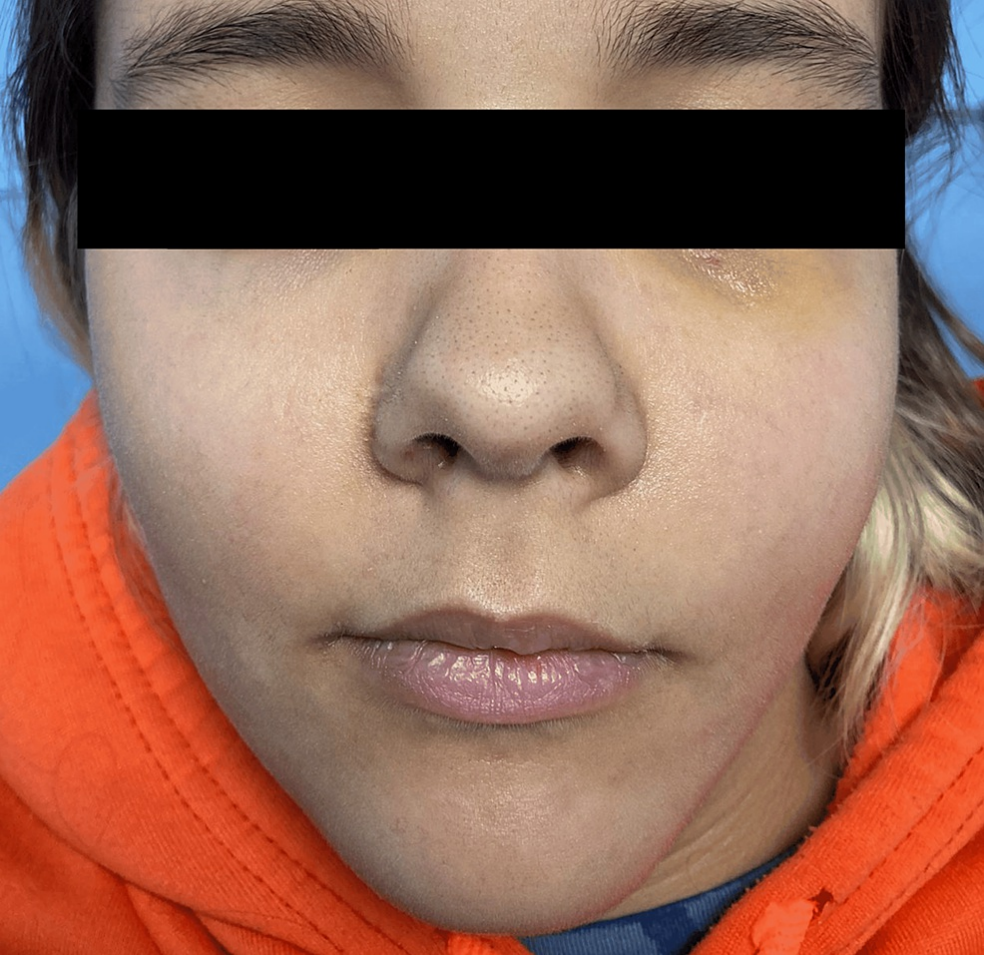
Two weeks post surgery Small bruise on the left infraorbital area and total resolution of the swelling.

Postoperative follow-up (two weeks): the patient had no complaints, and the incisions were healed (Figure 7).

**Figure 8:**
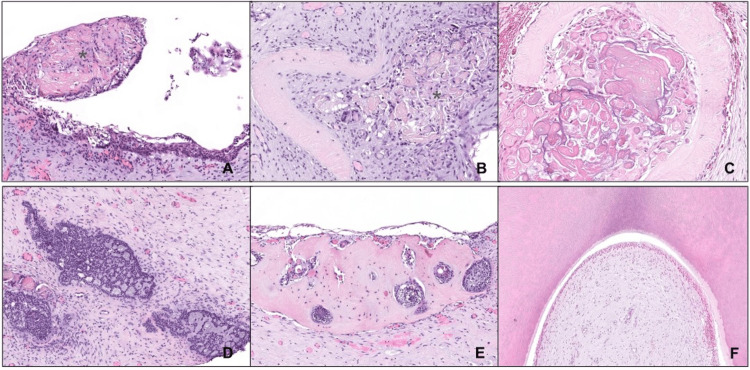
Histologic features of calcifying odontogenic cyst A: The cyst was lined by stratified squamous epithelium; foci of luminal proliferations including “ghost cells” were observed (*) (H&E, 200x). B: Aggregation of ghost cells in the pericystic connective tissue, associated with foreign-body granulomatous inflammation (*) and deposition of dentinoid matrix (H&E, 400x). C: Masses of ghost cells and dentinoids were found within the cyst wall (H&E, 520x). D: Focal stromal proliferation of ameloblastic-like epithelium, with characteristic palisaded and hyperchromatic columnar basal cells, disclosing cribiform/adenoid pattern (200x). E: Ameloblastic-like areas in relation to dentinoid matrix (200x). F: There was an associated odontoma (200x).

Pathological examination showed features of COC (Figure 8). The cyst was lined by stratified squamous epithelium with ameloblastic-like features, disclosing palisaded hyperchromatic basal cells and loosely arranged suprabasal cells. Numerous groups of ghost cells were observed, with abundant eosinophilic cytoplasm and loss of cell nucleus. These groups were identified in luminal and in stromal locations and occasionally presented dystrophic calcification. Abundant eosinophilic dentinoid-like matrixes were observed in the stroma, underlying foci of odontogenic epithelium. Moreover, focal proliferation of odontogenic ameloblastic such as epithelium with cribriform/adenoid pattern was identified in the stroma. In a luminal location, a lesion with abundant tubular dentine and ameloblastic epithelium was identified, with features compatible with an odontoma. By immunohistochemistry, abnormal nuclear expression of beta-catenin was noticed in the lesional cells. No expression of the BRAF p.V600E mutant protein was observed.

## Discussion

Hybrid odontogenic lesions, such as the entity in our work, are defiant for both pathologists and surgeons because of their controversial histogenesis and poorly understood clinical evolution. These lesions combine the histological characteristics of two or more odontogenic tumors, or individual cysts, in the same location, posing a significant challenge to medical professionals. Among hybrid odontogenic lesions, COCs associated with odontoma are the most common, accounting for 18.2% of all cases [4,5].

As mentioned, the nomenclature of COC-type lesions has been the subject of several revisions given their unknown histogenesis. Recently, mutations in the CTNNB1 gene were identified, which led to changes in the encoding of ß-catenin [4]. COCs can be classified into four subtypes: simple cysts, associated with odontoma, proliferative ameloblastomatous, and associated with other nonodontoma benign odontogenic lesions [6]. The subtype associated with odontoma represents 20-24% of all COC and manifests itself at an early age, on average, of 17 years [6].

Regarding its pathogenesis, two hypotheses are currently considered. The first reiterates the development of COC from the odontogenic epithelium forming the odontoma and the second argues that the odontogenic epithelium has the potential to differentiate into mesenchyme, leading to the secondary formation of the odontoma [6]. Additional studies are needed to better clarify this issue.

The differential diagnosis of this type of lesion includes the following entities: unicystic ameloblastoma, adenoid ameloblastoma, dentinogenic ghost cell tumor, and odontogenic ghost cell carcinoma. As previously described, the COC has equal gender prevalence, manifesting mainly in the second and third decades of life and in the anterior region of the maxilla [1,7].

Histologically, a cystic architecture is described, with a high number of ghost cells [7,8]. Both unicystic ameloblastoma and adenoid ameloblastoma have a higher prevalence in males and a very characteristic "ameloblastoma-like" epithelium. However, the first manifests itself earlier, in the second decade of life, presents a cystic cellular architecture, and is located, in most cases, in the posterior portion of the body of the mandible. The second manifests itself in the fourth decade of life, presents a cribriform architecture, and with no location of greater prevalence [1,9]. Dentinogenic ghost cell tumor and odontogenic ghost cell carcinoma both present histological features overlapping with COC, ghost-like cells, and ameloblastoma or "ameloblastoma-like" epithelium. However, ghost cell odontogenic carcinoma is a malignant neoplasm and ghost cell dentinogenic tumor, although a benign entity is a solid tumor with the presence of abundant dentinoid tissue, which differentiates it from COC [4,7].

Despite similarities with regular odontogenic cysts, this condition has a rapid growth rate and can deform adjacent structures. This is particularly concerning in pediatric patients whose bones are still growing as it can lead to bone deformities. COC is a rare entity among odontogenic cysts, constituting less than 1%. Despite the presence of ameloblastic proliferation in the histopathological analysis, the prognosis is generally good, and the progression to malignancy is an uncommon occurrence [3]. Recurrence rates are less than 5% and typically present within the first five years. Therefore, long-term follow-up, of at least 10 years, is advised to ensure early detection of any possible recurrence [4,9].

## Conclusions

In conclusion, this uncommon entity among head and neck tumors has a benign behavior; however, it can expand, reach large dimensions, and affect vital organs such as the eye. Despite the lack of consensus on the best surgical approach and follow-up plan, the established therapeutic strategy with enucleation of the lesion and bone curettage has demonstrated significant success. Its uniqueness provides an opportunity for in-depth study and analysis, which could pave the way for improved understanding and reinforce the management of similar cases.
